# Intraoperative coagulopathy during cesarean section as an unsuspected initial presentation of COVID-19: a case report

**DOI:** 10.1186/s12884-020-03140-2

**Published:** 2020-08-24

**Authors:** Kelly Elizabeth Kinsey, Eric Ganz, Susan Khalil, Lois Brustman

**Affiliations:** 1 Department of Obstetrics and Gynecology, Icahn School of Medicine at Mount Sinai West, 1000 10th Avenue, NY New York, USA; 2 Division of Minimally Invasive Surgery, Department of Obstetrics and Gynecology, Icahn School of Medicine, New York, USA; 3 Division of Maternal Fetal Medicine, Department of Obstetrics and Gynecology, Icahn School of Medicine at Mount Sinai West, New York, USA

**Keywords:** Sars-CoV-2, COVID-19, cesarean section, coagulopathy, D-Dimer

## Abstract

**Background:**

The world’s understanding of COVID-19 continues to evolve as the scientific community discovers unique presentations of this disease. This case report depicts an unexpected intraoperative coagulopathy during a cesarean section in an otherwise asymptomatic patient who was later found to have COVID-19. This case suggests that there may be a higher risk for intrapartum bleeding in the pregnant, largely asymptomatic COVID-positive patient with more abnormal COVID laboratory values.

**Case:**

The case patient displayed D-Dimer elevations beyond what is typically observed among this hospital’s COVID-positive peripartum population and displayed significantly more oozing than expected intraoperatively, despite normal prothrombin time, international normalized ratio, fibrinogen, and platelets.

**Conclusion:**

There is little published evidence on the association between D-Dimer and coagulopathy among the pregnant population infected with SARS-CoV-2. This case report contributes to the growing body of evidence on the effects of COVID-19 in pregnancy. A clinical picture concerning for intraoperative coagulopathy may be associated with SARS-CoV-2 infection during cesarean sections, and abnormal COVID laboratory tests, particularly D-Dimer, may help identify the patients in which this presentation occurs.

## Background

With the spread of SARS-Cov-2, lessons learned both locally and globally are providing us with the knowledge to arrive at evidence-based practices in this unforeseen pandemic. This case study discusses the possibility of SARS-CoV-2 instigating an abnormal hemodynamic response and coagulopathy in a patient undergoing a primary cesarean section in the setting of abnormal COVID laboratory values and normal coagulation factors.

## Case Presentation

This case describes a 26 year-old G1P0 who presented to a large Manhattan hospital at 37 weeks 6 days for a scheduled external cephalic version. Her past medical history was notable for well-controlled chronic Hepatitis B with an undetectable viral load (< 10 IU/mL) and normal liver function tests. In triage, she was found to have oligohydramnios and a confirmed frank breech presentation. Accordingly, delivery by cesarean section was recommended. As per routine practice in this hospital, the patient was tested for SARS-CoV-2 in triage. She denied symptoms of the disease at the time, was clinically perceived to be asymptomatic, and had normal vitals and a benign physical exam. While in triage, she began to have regular contractions on tocometer, so the decision was made to proceed with the cesarean section prior to obtaining results of the COVID testing. She was treated as a “person under investigation” (PUI) and healthcare personnel took the recommended precautions, including donning the appropriate personal protective equipment, having a more senior resident with an attending perform the cesarean section, and proceeding with spinal anesthesia to avoid aerosolizing procedures. Laboratory values prior to surgery inclusive of platelets (257 K/µL), prothrombin time (13.4 s), and international normalized ratio (1.0) were reported as normal (Table 1). Per routine protocol, she was type and crossed upon admission, with whole blood available upon request from the blood bank.

**Table 1:** Preoperative and postoperative laboratory values

	Preoperative		Postoperative Day 1	
	Value	Units	Value	Units
WBC	11.3	K/µL	19.8	K/µL
HCT	29.5	%	27.6	%
Hb	9.5	g/dL	9.3	g/dL
PLT	257	K/µL	246	K/µL
D-Dimer	-	-	19.1	µg/mL
LDH	-	-	271	U/L
CRP	-	-	12.24	mg/dL
PT	13.4	seconds	13.3	seconds
INR	1.0		1.0	
Fibrinogen	-	-	399	mg/dL

From the start of the case, there was more than an expected amount of bleeding noted, beginning from the insertion of the spinal needle and continuing through the initial incisions through the subcuticular and subcutaneous layers. The cesarean section proceeded in routine fashion. Following delivery of the fetus, uterine atony was noted with moderate bleeding, which persisted after administration of oxytocin and closure of the hysterotomy. Given that the patient had no history of elevated blood pressures and perioperatively had blood pressures of 90's/40's, the decision was made to administer 0.2 mg IV methylergonovine. A second dose was later administered for intermittent uterine atony after 30 min, with significant improvement in uterine tone. Hemostasis at the hysterotomy was confirmed and the muscle and fascia were closed. Just prior to closure of the subcuticular layer, it was necessary to cauterize multiple bleeding capillaries within the subcutaneous layer to achieve hemostasis. The subcuticular layer was closed with 4 − 0 vicryl. At this time, significant and persistent oozing was noted from the skin incision. Pressure was applied for several minutes with an improvement and then a tight pressure dressing was placed. During the case, administration of tranexamic acid was considered given significant intraoperative oozing; however, the decision was made to expectantly monitor the patient, given her unknown COVID status and the possible risk of exacerbating an existing hypercoagulable state. After several hours of monitoring, the patient had no further active bleeding from the abdominal incision and her vaginal bleeding was minimal. The total estimated blood loss was 1000 mL and her postoperative hematocrit was 27.6%.

Approximately 8 h after her surgery, nasopharyngeal testing by PCR resulted positive for SARS-CoV-2. Upon further questioning, the patient endorsed that she had been experiencing a very mild cough for several days prior to presentation that she did not find significant nor bothersome; she was not experiencing symptoms during her admission. Labs were drawn in response to the positive COVID testing postoperatively and reflected abnormal COVID labs in the setting of relatively normal coagulation factors. Specifically, postoperative laboratory values revealed an elevated D-Dimer, elevated LDH, elevated CRP, normal PT/INR, and normal fibrinogen values (Table 1). While D-Dimer, CRP, and LDH elevations can be expected in the setting of inflammation and tissue damage, as one may expect post-operatively, the increase in D-Dimer was significantly higher than what has been noted from patients postoperatively after cesarean section at this hospital. D-Dimer levels have generally been observed to range from approximately 2–6 µg/mL among the population referenced at this time of this publication, while this patient’s post-operative D-Dimer was 19.1 µg/mL – a notable difference.

The patient remained stable and afebrile, and she was ultimately discharged on postoperative day two in excellent condition, asymptomatic, with pain well-controlled, and meeting all postoperative milestones. Upon postpartum evaluation by telehealth several days after discharge, the patient reported that she was recovering well without concerns, and she was asymptomatic without any further symptoms of COVID-19.

## Discussion and Conclusions

This case describes a scenario in which a patient had abnormal intraoperative oozing in the face of a retrospectively-positive test for SARS-CoV-2, no symptoms of COVID-19 at the time of initial presentation, and acutely abnormal COVID lab results (including abnormal D-Dimer, troponins, lactate dehydrogenase, and C-reactive protein), in the setting of normal coagulation factors (prothrombin time, international normalized ratio, fibrinogen) and platelets.

While it is now widely accepted that SARS-CoV-2 induces a hypercoagulable state and produces an elevated risk of thrombosis,[1–3] there is emerging literature in the non-pregnant population to suggest that this virus is also associated with an elevated hemorrhage risk. Case reports have documented such findings as a hemorrhage within the basal ganglia and upper gastrointestinal bleeds among patients with COVID.[4, 5] Another study discusses hemorrhage and petechiae as an initial presentation of COVID, leading providers to initially misdiagnose the patient with dengue.[6] It is possible that COVID may likewise present in the form of an intrapartum coagulopathy, as suggested by the persistent oozing in this case. This presentation is particularly concerning in the setting of normal results for routine coagulation testing prior to surgery, which we may look toward to identify a coagulopathy preoperatively.

Additionally of note was the elevated D-Dimer in this case (19.1 µg/mL), which was elevated beyond what has been typically observed among this hospital’s COVID-positive peripartum pregnant population. D-Dimer is produced when plasmin degrades cross-linked fibrin, and accordingly is a marker of both an active coagulation process as well as fibrinolysis. Unsurprisingly, D-Dimer has been found to be associated with hemorrhage in prior studies.[7] Recent studies in the non-pregnant population have also found elevated D-Dimer, fibrinogen, and fibrinogen degradation product values among patients infected with SARS-CoV-2, along with decreased anti-thrombin levels, and have found these abnormalities to have prognostic value on mortality among patients infected with SARS-CoV-2.[8] There may exist a spectrum upon which abnormal bleeding will present depending upon the severity of the COVID infection, particularly in relation to abnormal COVID laboratory markers. We are suggesting that there may be a higher risk for intrapartum bleeding in the pregnant, COVID-positive patient who displays more aberrant COVID laboratory values.

Given the novel nature of SARS-CoV-2, this case presents the important finding of an unexpected initial presentation of COVID-19. Ultimately, further research is needed to assess whether pronounced oozing that is clinically concerning for a coagulopathy is a common presentation of COVID-19 in the parturient patient. It is possible that standard COVID laboratory tests, and particularly D-Dimer, should be routinely drawn among “persons under investigation” and COVID-positive patients preoperatively and at delivery, with additional hemorrhage precautions taken in patients with unexpectedly abnormal values. However, future studies are needed to answer these questions. The knowledge we gain will further inform the management of COVID-positive pregnancies and, in this case, the intersection between COVID19 and cesarean sections.

## Data Availability

Data sharing is not applicable to this article as no datasets were generated or analyzed during the current study.

## Abbreviations

COVID-19: Coronavirus disease 2019
SARS-CoV-2: Severe acute respiratory syndrome coronavirus 2
AFI: Amniotic fluid volume
PUI: Persons under investigation (for COVID-19 disease)
PCR: Polymerase chain reaction
WBC: White blood cell (count)
HCT: Hematocrit
Hb: Hemoglobin
PLT: Platelets
LDH: Lactate dehydrogenase
CRP: C reactive protein
PT: Prothrombin time
INR: International normalized ratio
